# Structure Matters: The Role of Clique Hierarchy in the Relationship Between Adolescent Social Status and Aggression and Prosociality

**DOI:** 10.1007/s10964-015-0310-4

**Published:** 2015-06-16

**Authors:** Kim Pattiselanno, Jan Kornelis Dijkstra, Christian Steglich, Wilma Vollebergh, René Veenstra

**Affiliations:** Interuniversity Center for Social Science Theory and Methodology, Department of Sociology, University of Groningen, Grote Rozenstraat 31, 9712 TG Groningen, The Netherlands; Department of Interdisciplinary Social Science, Utrecht Centre of Child and Adolescent Studies, Utrecht University, Heidelberglaan, 1, 3584 CS Utrecht, The Netherlands

**Keywords:** Cliques, Hierarchy, Peer status, Popularity, Aggression, Prosocial behavior

## Abstract

Peer cliques form an important context for the social development of adolescents. Although clique members are often similar in social status, also within cliques, status differences exist. How differences in social status between clique members are related to behaviors of its individual members is rather unknown. This study examined to what extent the relationship of individual social status (i.e., perceived popularity) with aggression and prosocial behavior depends on the level of internal clique hierarchy. The sample consists of 2674 adolescents (49.8 % boys), with a mean age of 14.02. We focused specifically on physical and relational aggression, and practical and emotional support, because these behaviors have shown to be of great importance for social relationships and social standing among adolescents. The internal status hierarchy of cliques was based on the variation in individual social status between clique members (i.e., clique hierarchization) and the structure of status scores within a clique (pyramid shape, inverted pyramid, or equal distribution of social status scores) (i.e., clique status structure). The results showed that differences in aggressive and prosocial behaviors were particularly moderated by clique status structure: aggression was stronger related to individual social status in (girls’) cliques where the clique status structure reflected an inverted pyramid with relatively more high status adolescents within the clique than low status peers, and prosocial behavior showed a significant relationship with individual social status, again predominantly in inverted pyramid structured (boys’ and girls’) cliques. Furthermore, these effects differed by types of gender cliques: the associations were found in same gender but not mixed-gender cliques. The findings stress the importance of taking into account internal clique characteristics when studying adolescent social status in relationship to aggression and prosociality.

## Introduction

During adolescence, peers become increasingly important for the social and emotional development of adolescents (Rubin et al. 2006). Reflecting this, adolescents spend a lot of their time with peers, particularly in smaller groups of friends, so-called peer cliques (Brown 2004; Gifford-Smith and Brownell 2003; Hallinan 1980; Salkind 2008), which become highly salient in early adolescence (Brown 2004; Steinberg and Monahan 2007). Adolescent peer cliques have been identified as a developmentally important unit of analysis (Adler and Adler 1998; Espelage et al. 2007) as they form a setting in which adolescents hang around, gain a sense of belonging and receive support (e.g., Ellis and Zarbatany 2007; Kwon and Lease 2007; Prinstein and La Greca 2002).

An important way to distinguish different types of adolescent peer cliques is to look at the average social status of the clique in the broader peer context (Adler and Adler 1998; Corsaro and Eder 1990). Attaining a high social status or becoming popular as an individual is important in adolescence (Buhrmester 1990; Cillessen and Rose 2005; Jarvinen and Nicholls 1996; Ojanen et al. 2005). Those with high social status can demonstrate power and influence over others and receive affection from others who also wish to have a high status themselves (Dijkstra et al. 2010; Merten 1997; Parkhurst and Hopmeyer 1998). Reflecting this, some cliques have a higher position in the status hierarchy than other cliques. Social status differences between cliques are accompanied by distinct characteristics and behaviors of members of those cliques, most prominently aggressive and prosocial behaviors (e.g., Adler and Adler 1998; Closson 2009; Garandeau et al. 2011). Both behaviors are found to be more pronounced in higher status cliques, reflecting the associations between social status among peers and aggression and prosocial behavior on the individual level (e.g., Cillessen and Rose 2005; Dijkstra et al. 2009; Ellis and Zarbatany 2007; Peters et al. 2010).

However, in assessing mean status differences *between* cliques, possible differences in social status between members *within* the same clique are ignored. Although members within cliques tend to be quite similar in individual social status (Cairns and Cairns 1995; Dijkstra et al. 2012; Kupersmidt et al. 1995), status differences can and do emerge also between individuals *within* cliques (e.g., Adler and Adler 1998; Closson 2009). Basically, some cliques may be more hierarchical with large differences in social status between clique members, whereas other cliques may be more egalitarian with small differences in social status between clique members. Although the importance of such differences within cliques has been acknowledged (Brown 1990), it remains unknown whether these differences might be related to behaviors of its individual members, as the internal hierarchy might steer distinct clique dynamics.

The aim of this study is, therefore, to examine structural differences between cliques in relationship to status and behaviors. More specifically, we examine to what extent the relationship between individual social status (i.e., perceived popularity) and behaviors that have been related to status, namely aggression (physical and relational aggression) and prosocial behavior (emotional and instrumental support) (Cillessen and Rose 2005; Mayeux and Cillessen 2008), depend on the internal status hierarchy within cliques. Status hierarchy within cliques is defined in two ways: the level of *clique hierarchization* and *clique status structure*. *Clique hierarchization* was based on the variation (i.e., standard deviation) in individual social status within cliques, with large differences indicating hierarchical cliques and small differences indicating egalitarian cliques. A similar approach has been used in previous research on hierarchies within classrooms (e.g., Garandeau et al. 2013; Zwaan et al. 2013). An important limitation of this measure is that it is less informative about the structure of the hierarchy. A clique could contain a “typical” top down hierarchical structure with a few individuals having a high status and many with a very low status (pyramid shape; as an illustration see Appendix Fig. 3a, b), but also an inverted pyramid (Appendix Fig. 3c). Furthermore, cliques could contain an equal distribution of low and high status individuals, and still display a high clique status standard deviation (Appendix Fig. 3d). Hence, these different configurations are not captured by the standard deviation for individual status within cliques. Therefore, we also consider *clique status structure* as a measure of hierarchy by subtracting the clique status median score from the mean, introducing a new measure of clique hierarchy which captures the above mentioned configurations. Specifically, positive values of this measure imply clique hierarchies with a pyramid shape, whereas negative values indicate an inverted pyramid with relatively more clique members having a high status than a low status.

## The Role of Status Differences within Cliques

The question is how status hierarchy within cliques might affect the relationship between social status and behaviors. Starting with aggressive behaviors, it has been argued that large status differences between individuals are related to a power imbalance, which in turn promotes aggression (Adler and Adler 1998; Wilkinson and Pickett 2009). The explanation is that individuals at the bottom of the status hierarchy are “easy” victims for higher status peers, who can exert their power upon those lower in status. A recent study by Garandeau et al. (2013), for example, showed that higher levels of classroom status hierarchy were associated with higher levels of bullying, a specific form of aggression among adolescents. In a similar way, Wolke et al. (2009) showed that it was more likely to be a victim of relational aggression in classrooms with a stronger hierarchy than in more egalitarian classrooms. Closson (2009) showed that aggression towards clique members was associated with a higher status in the clique, and those who were more dominant used more overt and relational forms of aggression. Furthermore, in another study Garandeau et al. (2011) showed that the positive relationship between an individual’s status and aggression was stronger in more hierarchical classrooms compared to more egalitarian classrooms. Together, these studies draw attention to possible negative consequences of a hierarchical ordering in peer groups as it seems to go together with aggressive behaviors.

Whereas previous studies assume that high status adolescents more easily display aggression towards low status children to emphasize their dominance, we believe that aggression should be considered in the light of its function to maintain social status. Although some adolescents value status more than others (LaFontana and Cillessen 2010; Ryan and Shim 2006), in general, adolescents do wish to increase their status position among peers (Lindenberg 1996). As status is a positional good implying that not everyone can have a high status, whereas at the same time people generally strive for status, the consequence is that individuals compete with each other for status. Such competition should specifically be pronounced in groups and contexts with small differences in status. Here, adolescents could be more aware of others who could challenge their position and compete with them for status (see also Adler and Adler 1998; Eder 1985). In order to maintain their social ranking, adolescents might be more inclined to display aggressive behaviors that reflect and emphasize a powerful and dominant position among peers (see also Cillessen and Mayeux 2004; Dijkstra et al. 2009). In reverse, in groups and contexts with large status differences, competition for status is less, and, hence, aggression is less needed to maintain status. Hierarchies can stabilize relationships and decreases hostility in groups (Pellegrini and Long 2002; Savin-Williams 1979), because individuals learn their position in the group and no longer compete for status (Hawley 1999). Zwaan et al. (2013) for example showed that status was more strongly related to aggression when status differences in classrooms were smaller. Building on this latter approach, we expect a stronger relationship between individual status and aggression when status differences between clique members are smaller (i.e., in more *egalitarian* cliques; Hypothesis 1a) and when the clique status structure reflects an inverted pyramid with relatively more high status adolescents within the clique than low status peers (Hypothesis 1b).

Furthermore, studies showed that high status adolescents are not only characterized by negative behaviors, such as aggression, but also by prosocial behavior (De Bruyn and Cillessen 2006; Dijkstra et al. 2009; Hymel et al. 2002). Prosocial behavior facilitates friendly relationships with peers (e.g., Asher and McDonald 2009; Buhrmester 1996; Closson 2009; Coie et al. 1990; Rubin et al. 2006), and might help high status adolescents to mitigate the negative effects of their aggressive behavior (De Bruyn and Cillessen 2006; Dijkstra et al. 2009). However, prosocial behavior towards others by providing support and aid also implies a certain risk because it is uncertain if and when prosocial acts will be reciprocated over time. Individuals will in most cases display prosocial behavior if they expect a similar act in return (Clark and Mils 1993). For that reason, prosociality is more likely to emerge among individuals who can rely on each other. However, in a competitive context, reciprocity is less certain and it can be costly to act prosocially (Clark and Mils 1993). Also, seeking help can be costly, because it exposes individuals’ weaknesses (Ryan et al. 2001; Shim et al. 2013), which can hinder both attainment and maintenance of status in a competitive context. Hence, it could be argued that prosociality will particularly flourish in situations where individuals do not compete with each other. This implies that the relationship between individual status and prosocial behavior is stronger in cliques with large status differences as there is less competition. Hence, we expect a stronger relationship between individual status and prosocial behavior when status differences between clique members are larger (i.e., in more *hierarchical* cliques) (Hypothesis 2a) and when the clique status structure reflects a pyramid with relatively less high status adolescents within the clique than low status peers (Hypothesis 2b).

## The Present Study

This study examines in what way the relationship between adolescents’ individual social status, and aggression and prosocial behavior varies by the internal clique hierarchy. Specifically, we examine the impact of social status differences *within* adolescent peer cliques, with clique hierarchization and clique status structure, on the relationship between individual social status and aggression (physical aggression and relational aggression) and prosocial behavior (emotional and instrumental support). Furthermore, gender will be taken into account, because boys are often more physically aggressive than girls, whereas girls are more relationally aggressive (Dijkstra et al. 2009; LaFontana and Cillessen 2002; Rose et al. 2004; Vaillancourt and Hymel 2006), and girls often display more prosocial behavior than boys (e.g., Maccoby and Jacklin 1974; Rose and Rudolph 2006). Moreover, we take into account the gender composition of the clique by testing our hypotheses for same-gender (either boys or girls) and mixed-gender cliques separately as boys and girls differ in their relationships. Boys’ relationships are often characterized by sharing mutual interests and girls’ relationships by intimacy and support, whereas relationships between boys and girls combine characteristics of both (e.g., McDougall and Hymel 2007). Furthermore, early adolescents prefer friendships with same-gender peers, but at the same time cross-gender friendships steadily increase (Maccoby 1990; Rose and Rudolph 2006).

## Methods

### Participants and Procedure

In the present study, we used a subsample of data from a large cohort study, TRAILS (TRacking Adolescents’ Individual Lives Survey, De Winter et al. 2005). TRAILS is a prospective cohort study of Dutch preadolescents who will be measured biennially until they are at least 25 years old. TRAILS is designed to chart and explain the development of mental health and social development from preadolescence into adulthood. Of all the children approached for enrollment in the study (selected by the municipalities and attending schools that were willing to participate; *N* = 3145 children from 122 schools; response of schools 90.4 percent), a total of 2230 children participated in the first assessment wave (T1) of TRAILS. Of the 2230 baseline participants, 96.4 % (*N* = 2149, 51.0 % girls) participated in the second assessment wave (T2).

In addition to the regular questionnaires, which were filled out by TRAILS participants only, the T2 assessment wave also included peer nominations, which were collected from both TRAILS participants and their classmates. Peer nominations were assessed by nominations of all classmates in classes with at least three regular TRAILS participants. Schools provided the names of classmates of TRAILS participants. All eligible students then received an information letter for themselves and their parents, in which they were asked to participate. If students or their parents wished to refrain from participation, they were requested to send a reply card within 10 days. In total, 98 students, of whom three were regular TRAILS participants, refused to participate. Approximately 2 weeks after the information letter had been sent, a TRAILS staff member visited the selected school classes to assess the peer nominations within class. The assessment of the peer nominations lasted about 15 min and took place during regular lessons. Peer nominations were assessed in a total of 172 classes in 34 schools in the first grade (72 school classes) and second grade (100 school classes) of secondary education, and were cued to peers in the same class. Of all 3672 children that were approached to participate in this study, 90.2 % filled out the questionnaire and nominated their classmates. This yielded a total number of 3312 students (1675 boys, 1637 girls), including 1007 regular TRAILS participants (*M* age = 14.02, *SD* = 0.73). Each classroom contained on average 18.39 participating pupils (*SD* = 5.99; range from 7 to 30).

### Measures

*Cliques* were identified based on the network of friendship nominations in each class. Adolescents could nominate an unlimited number of friends within the class. A relationship was considered if at least one person indicated that they were friends. Hence, nominations did not need to be mutual. Following the two-step method of *clique overlap analysis* (Borgatti et al. 2013; Everett and Borgatti 1998), we first identified groups of size three or higher in which everyone was connected to everybody else (graph-theoretical cliques). This set of partly overlapping groups was used to construct a matrix of proximity scores, indicating for each pair of individuals the number of groups they jointly are part of. Second, based on a hierarchical clustering of this proximity matrix (Johnson 1967), non-overlapping groups were identified, which we used to operationalize the cliques in this article. Average clique size and the proportion of individuals allocated to a clique increased with decreasing proximity level at which this clustering process was evaluated. We chose as cutoff a proximity level where, out of the total sample (*N* = 3312), more than 80 % of all individuals were assigned to a clique with a minimum of three members. This ultimately led to 534 identified cliques containing 2674 adolescents (M/F = 1331/1343) and a distribution of clique sizes (*M* = 5.72, SD = 2.24) dovetailing with earlier research on adolescent cliques (Salkind and Rasmussen 2008). We are aware of the multitude of alternative algorithms to extract cliques from network data (Fortunato 2010; Porter et al. 2009), but are confident that these would not have resulted in very different groups.

Because in this study we focused on clique characteristics, our target sample only includes participants residing in cliques (*N* = 2674). Participants who did not belong to a clique were on average lower in status, more physically aggressive, displayed less relational aggression, and gave less emotional and instrumental support according to their classmates (see Appendix 2).

*Individual social status* In order to determine *individual status* among peers, individual proportion scores were calculated within cliques (not class) for the number of nominations received on the question “Who do others want to be associated with?”. Next, proportion scores were calculated by dividing the total number of nominations received by the number of nominating clique members. Because we are interested in the internal clique dynamics, we focused on nominating clique members and not all classmates. This yielded a measure of adolescents’ individual status, ranging from 0 (low status) to .80 (high status). We explicitly disentangled personal preferences for being associated with a person from reputation-based preferences by asking respondents to nominate people with whom others want to be associated with, instead of who they themselves want to be associated with. We believe that this yielded a reputation-based measure for social status. This question has been used in previous research, showing similar associations with other peer status measures (e.g., acceptance, rejection) and behaviors compared to studies using most and least popular peer nominations (e.g., Dijkstra et al. 2008, 2009, 2010).

*Clique status* In order to determine *clique status* (i.e., the overall status of a clique in the larger peer context), we calculated a mean score of individual status proportion scores for each clique. This yielded a continuous measure for clique status, ranging from 0 (low status clique) to .56 (high status clique).

*Clique size* Clique size was determined by the number of clique members in an adolescent’s clique (including the adolescent him- or herself). This resulted in an average clique size of 5.72 (range from 3 to 18).

*Clique hierarchization* To assess whether a clique was more hierarchical (i.e., had large differences in individual status scores) or more egalitarian (i.e., had small differences in individual status scores), we first calculated a continuous measure of *clique hierarchization* based on the standard deviation of individual status proportion scores within the clique. By examining the distance between individuals in a clique, we are able to approximate how clique members relate to one another. This serves as an indicator for the presence (or absence) of a hierarchy (see also Garandeau et al. 2013; Zwaan et al. 2013). A large standard deviation in proportion scores indicates a more hierarchical clique, reflected by larger differences in individual status within that clique, whereas more egalitarian cliques have a smaller standard deviation. This yielded a hierarchization score for cliques, running from 0 to .39 (*M* = .10, *SD* = .08).

*Clique status structure* We measured *clique status structure* by subtracting for each clique the median score from the mean of individual status proportion scores (clique status). This resulted in a measure of hierarchy structure ranging from −.24 to .28 (*M* = .02, *SD* = .06), where positive scores indicate that more individuals reside at the bottom of a clique (pyramid) and negative scores indicate that more individuals reside at the top of a clique (inverted pyramid). Scores approaching zero indicate an equal distribution of low and high status peers balance in the clique.

*Aggression* We used physical aggression and relational aggression, derived from peer nominations, as measures for *aggression*. Students could nominate their classmates on the items “Who quarrels and/or initiate fights often?” to assess physical aggression, and “Who spreads gossip/rumors about others?” to assess relational aggression. Proportion scores were calculated by dividing the total number of nominations received by the number of nominating clique members (again, not classmates), yielding scores from 0 to .86 for physical aggression, and 0 to .83 for relational aggression. Physical aggression and relational aggression correlated .16, and individual status correlated positively with both forms of aggression (respectively; *r* = .16; *r* = .20).

*Prosocial behavior* We measured *prosocial behavior* using peer nominations for the questions “Which classmates give you emotional support when you are despondent (e.g., problems at home)?” (emotional support) and “Which classmates give you practical support (e.g., with homework)?” (instrumental support). Proportion scores were calculated for emotional support (ranging from 0 to .89), and instrumental support (ranging from 0 to .88), by dividing the total number of nominations received by the number of nominating clique members. Emotional and instrumental support correlated .60, and individual status correlated positively with both forms of prosociality (respectively; *r* = .12 and *r* = .07).

### Analyses

Although the data are classroom-based, we did not consider the class level in the description of the main variables as there was no variability at the class level. We used multilevel analysis with MlwiN 2.23 (Rasbash et al. 2009) to test our hypotheses. This way, we could account for non-independence of observations, caused by the nested structure of the data. We conducted the analysis with a two-level structure for boys’ cliques, girls’ cliques, and mixed gender cliques separately, with individuals (level one) nested in cliques (level two). On the individual level, we included the effect of gender as a control variable for mixed-gender cliques (girls = 0 and boys = 1). On the clique level we controlled for clique status and clique size. All predictor variables (except gender) were centered around the grand mean. Multilevel analyses were conducted in three steps. First, we assessed the effect of gender (for mixed-gender cliques). In the following step (models 1) individual status, clique status, clique size, and clique hierarchization/clique status structure were added to the model. Finally (models 2), we examined the interaction between individual status and clique hierarchization/clique status structure in the analyses to test our hypotheses.

## Results

### Descriptive Statistics

Although most adolescent peer cliques were of the same gender (*N* = 421), there was also a fair percentage (21 %) of mixed-gender cliques (*N* = 113) (Table 1). When comparing boys’ cliques, girls’ cliques, and mixed-gender cliques, it appeared that in mixed-gender cliques individual status was higher (according to clique members), mixed-gender cliques had a higher overall status, and were larger in size than same-gender cliques. Boys’ cliques were slightly larger than girls’ cliques. Furthermore, mixed-gender cliques were more hierarchical than same-gender cliques, but similar in clique status structure, and boys’ cliques had a somewhat more hierarchical structure than girls’ cliques. With regard to the outcome variables, it appeared that boys were more physically aggressive, whereas girls scored higher on relational aggression, particularly in mixed-gender cliques. Furthermore, both emotional and instrumental support was higher for girls than for boys, and among girls higher in same gender cliques than in mixed-gender cliques.

**Table 1:** Descriptive statistics of the sample split by clique gender composition

	Boys’ cliques	Girls’ cliques	Mixed-gender cliques		Difference		
	(*N* = 1012; *N*_cliques_ = 207)	(*N* = 976; *N*_cliques_ = 214)	Boys in mixed (*N* = 331; *N*_cliques_ = 113)	Girls in mixed (*N* = 355; *N*_cliques_ = 113)			
	Mean (*SD*)	Mean (*SD*)	Mean (*SD*)	Mean (*SD*)	*F*	*df*1, *df*2	*p*
Individual status	.09 (.15)^a^	.10 (.17)^a^	.14 (.16)^b^	.14 (.17)^b^	11.45	3,2670	<.01
Clique status	.09 (.10)^a^	.10 (.12)^a^	.14 (.11)^b^		23.96	3,2670	<.01
Clique size	5.46 (1.79)^b^	5.07 (1.52)^a^	7.04 (2.90)^c^		127.14	3,2670	<.01
Clique hierarchization	.09 (.08)^a^	.09 (.09)^a^	.12 (.07)^b^		19.83	3,2670	<.01
Clique status structure	.025 (.050)^b^	.016 (.058)^a^	.017 (.060)^ab^		4.70	3,2670	<.01
Physical aggression	.09 (.17)^b^	.03 (.09)^a^	.11 (.18)^c^	.03 (.09)^a^	58.09	3,2670	<.01
Relational aggression	.06 (.13)^a^	.10 (.16)^b^	.09 (.15)^b^	.18 (.20)^c^	46.42	3,2670	<.01
Emotional support	.28 (.22)^a^	.50 (.23)^c^	.24 (.19)^a^	.40 (.23)^b^	203.43	3,2670	<.01
Instrumental support	.37 (.23)^a^	.54 (.21)^c^	.35 (.22)^a^	.40 (.21)^b^	119.98	3,2670	<.01

Means in the same row that do not share superscripts differ at *p* < .05 in the Bonferroni test

Correlations were calculated for boys and girls separately within same- and mixed-gender cliques (see Appendices 3 and 4). Positive correlations were found between individual social status and aggression/prosocial behavior in all clique types (ranging from *r* = .07, *p* < .05 to *r* = .36, *p* < .01), except for instrumental support in girls’ cliques and girls in mixed-gender cliques. Physical and relational aggression were positively related (ranging from *r* = .14 to .31, *p* < .01), and emotional and instrumental support correlated positively in all clique types (ranging from respectively *r* = .14 to .31, *p* < .01, and *r* = .47 to .54, *p* < .01). In boys’ cliques physical aggression and prosocial behavior showed a negative correlation (*r* = -.08, *p* < .01, and −.11, *p* < .01), and for girls in mixed-gender cliques physical aggression and instrumental support showed a negative significant correlation (*r* = −.26, *p* < .01). Furthermore, positive correlations were found in same gender, but not mixed-gender cliques for clique status and clique size (*r* = .12, *p* < .01), clique size and clique hierarchization (*r* = .25, *p* < .01, and *r* = .11, *p* < .01), and clique hierarchization and clique status structure (*r* = .50, *p* < .01, and *r* = .30, *p* < .01). Clique status was also positively related to clique hierarchization in all clique types (ranging from *r* = .71 to .78, *p* < .01), positively related to clique status structure in boys’ cliques (*r* = .11, *p* < .01), and negatively related to clique status structure in mixed-gender cliques (*r* = −.24 and −.28, *p* < .01). Clique size was positively related to clique status structure in boys’ and mixed-gender cliques (respectively *r* = .14 and .15, *p* < .01).

### Hypothesis Testing

#### Aggression

First we tested our hypothesis regarding aggression. For the interpretation of the results it should be kept in mind that the dependent variable has a range of 1, which results in relatively small regression coefficients. We first discuss the models with clique hierarchization and then the models with clique status structure. As shown in Table 2, there was a positive relationship between gender and physical aggression and a negative relationship between gender and relational aggression in mixed-gender cliques (Models 1), indicating that boys were more physically aggressive but less relationally aggressive than girls. Clique status was positively related to physical aggression in boys’, girls’, and mixed-gender cliques, and positively related to relational aggression in mixed-gender cliques. Clique size had a slight negative relationship with relational aggression in boys’ and mixed-gender cliques, and a slight positive relationship with relational aggression in girls’ cliques. Looking at the main effect of individual status, it appeared that status had a significant positive relationship with all types of aggression across all clique types, indicating that adolescents of higher status were more physically and relationally aggressive. Clique hierarchization had a positive main effect on relational aggression in boys’ cliques. To test our hypotheses, we examined the interaction effect between individual status and clique hierarchization (Models 2). Only for physical aggression in girls’ cliques a significant moderating effect was found of clique hierarchization.

**Table 2:** Multilevel models of individual status and clique hierarchization for physical and relational aggression

	Boys’ cliques				Girls’ cliques				Mixed-gender cliques^a^			
	Physical aggression		Relational aggression		Physical aggression		Relational aggression		Physical aggression		Relational aggression	
	Model 1	Model 2	Model 1	Model 2	Model 1	Model 2	Model 1	Model 2	Model 1	Model 2	Model 1	Model 2
	*b* (*SE*)	*b* (*SE*)	*b* (*SE*)	*b* (*SE*)	*b* (*SE*)	*b* (*SE*)	*b* (*SE*)	*b* (*SE*)	*b* (*SE*)	*b* (*SE*)	*b* (*SE*)	*b* (*SE*)
*Level 1*												
Individual status (IS)	.12* (.05)	.09 (.08)	.09** (.04)	.10 (.06)	.09*** (.02)	−.02 (.04)	.10* (.04)	.06 (.07)	.19*** (.04)	.24*** (.05)	.19*** (.04)	.20*** (.06)
*Level 2*												
Clique status	.26** (.08)	.26** (.08)	−.09 (.06)	−.09 (.06)	.14*** (.04)	.14*** (.04)	.06 (.07)	.06 (.07)	.17* (.09)	.18* (.09)	.33** (.11)	.33** (.11)
Clique size	−.00 (.01)	−.00 (.01)	−.01** (.00)	−.01** (.00)	.00 (.00)	.00 (.00)	.01** (.00)	.01** (.00)	.00 (.00)	.00 (.00)	−.01** (.00)	−.01** (.00)
Clique hierarchization (CH)	−.05 (.11)	−.05 (.11)	.41*** (.08)	.41*** (.08)	−.02 (.05)	−.02 (.05)	.07 (.09)	.07 (.09)	−.11 (.13)	−.11 (.13)	.26 (.17)	.26 (.17)
*Cross*-*level interaction*												
IS × CH		.33 (.70)		.02 (.53)		.90** (.29)		.36 (.50)		−.65 (.44)		.11 (.46)
*df*	4	1	4	1	4	1	4	1	4	1	4	1
χ^2^ Deviance difference^b^	21.54***	.22	86.51***	.01	40.71***	9.58**	27.34***	.51	28.31***	2.17	54.38***	.05

^+^ *p* < .1; * *p* < .05; ** *p* < .01; *** *p* < .001

^a^For mixed-gender cliques we also included gender as control variable (girl = 0/boy = 1). The effect of gender was .08*** (.01) for the models with physical aggression and −.08*** (.01) for the models with relational aggression

^b^The decrease in χ^2^ deviance for models 1 of boys’ and girls’ cliques is compared with the deviance of the empty model, and of mixed-gender cliques compared with the deviance of the model including only gender. Model 2 is compared with model 1

**Fig. 1:**
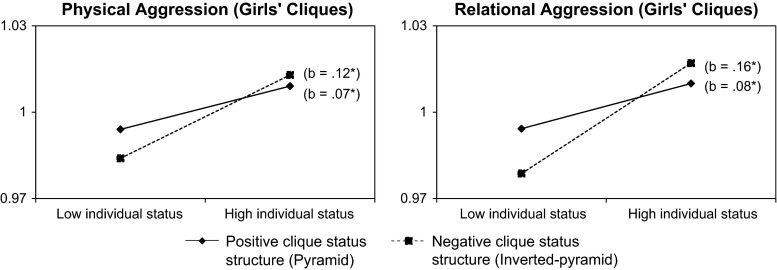
Simple slopes between individual status and physical aggression and relational aggression in girls’ cliques for positive and negative clique status structure (*p < .05)

Models with clique status structure were similar to those with clique hierarchization (see Models 1), with the exception that clique status was also positively related to relational aggression in girls’ cliques, and clique size only had a small positive relationship with relational aggression in girls’ cliques (Table 3). Furthermore, we found a negative main effect of clique status structure in mixed-gender cliques for both physical and relational aggression, suggesting that aggression was higher in cliques where the clique status structure reflected an inverted pyramid (i.e., with relatively more high status adolescents within the clique than low status adolescents). Regarding our hypotheses, a negative moderating effect of clique status structure was found on the relationship between individual status and aggression (both physical and relational aggression) in girls’ cliques (Model 2), indicating that individual status was particularly related to aggression in girls’ cliques with an inverted pyramid structure (i.e., with more high status adolescents in the clique than low status) (see Fig. 1).

**Table 3:** Multilevel models of individual status and clique status structure for physical and relational aggression

	Boys’ cliques				Girls’ cliques				Mixed-gender cliques^a^			
	Physical aggression		Relational aggression		Physical aggression		Relational aggression		Physical aggression		Relational aggression	
	Model 1	Model 2	Model 1	Model 2	Model 1	Model 2	Model 1	Model 2	Model 1	Model 2	Model 1	Model 2
	*b* (*SE*)	*b* (*SE*)	*b* (*SE*)	*b* (*SE*)	*b* (*SE*)	*b* (*SE*)	*b* (*SE*)	*b* (*SE*)	*b* (*SE*)	*b* (*SE*)	*b* (*SE*)	*b* (*SE*)
*Level 1*												
Individual status (IS)	.14** (.04)	.13** (.05)	.09** (.03)	.08** (.03)	.09*** (.02)	.10*** (.02)	.10* (.04)	.12** (.04)	.19*** (.04)	.19*** (.04)	.20*** (.04)	.20*** (.06)
*Level 2*												
Clique status	.22** (.07)	.22** (.07)	.11 (.06)	.11 (.06)	.13*** (.03)	.13*** (.03)	.10** (.05)	.10** (.05)	.16** (.06)	.16** (.06)	.53*** (.08)	.53*** (.08)
Clique size	−.00 (.00)	−.00 (.00)	−.00 (.00)	−.00 (.00)	.00 (.00)	.00 (.00)	.01*** (.00)	.01*** (.00)	.00 (.00)	.00 (.00)	−.01 (.00)	−.01 (.00)
Clique status structure (CSS)	−.22 (.15)	−.22 (.15)	.23^+^ (.13)	.23^+^ (.13)	.03 (.05)	.03 (.05)	.03 (.10)	.04 (.10)	.26* (.11)	.26* (.11)	.43** (.15)	.43** (.15)
*Cross*-*level interaction*												
IS × CSS		.50 (.65)		.31 (.45)		−.53* (.24)		−.86* (.41)		−.01 (.37)		−.41 (.38)
*df*	4	1	4	1	4	1	4	1	4	1	3	1
χ^2^ Deviance difference^b^	62.51***	.60	192.47***	.48	30.80***	4.70*	26.96***	4.31*	33.27***	.00	70.46***	1.13

^+^ *p* < .1; * *p* < .05; ** *p* < .01; *** *p* < .001

^a^For mixed-gender cliques we also included gender as control variable (Girl = 0/Boy = 1). The effect of gender was .08*** (.01) for the models with physical aggression and −.08*** (.01) for the models with relational aggression

^b^The decrease in χ^2^ deviance for models 1 of boys’ and girls’ cliques is compared with the deviance of the empty model, and of mixed-gender cliques compared with the deviance of the model including only gender. Model 2 is compared with model 1

#### Prosocial Behavior

With regard to prosocial behavior, we found that boys in mixed-gender cliques gave less emotional and instrumental support than girls in mixed-gender cliques (Models 1; Table 4). The relationship between clique status and emotional support was positive for girls’ cliques. No relationships were found between clique status and instrumental support in any type of clique. Furthermore, there was a small negative effect of clique size for emotional support in boys’ cliques. Also for prosocial behavior, individual status showed a positive main effect for all clique types, meaning that adolescents of higher status gave more emotional and instrumental support than adolescents of lower status. Clique hierarchization showed a negative relationship with emotional support in girls’ cliques. It appeared that clique hierarchization did not moderate the effect of individual status on emotional support and instrumental support in any type of clique (Models 2).

**Table 4:** Multilevel models of individual status and clique hierarchization for emotional and instrumental support

	Boys’ cliques				Girls’ cliques				Mixed-gender cliques^a^			
	Emotional support		Instrumental support		Emotional support		Instrumental support		Emotional support		Instrumental support	
	Model 1	Model 2	Model 1	Model 2	Model 1	Model 2	Model 1	Model 2	Model 1	Model 2	Model 1	Model 2
	*b* (*SE*)	*b* (*SE*)	*b* (*SE*)	*b* (*SE*)	*b* (*SE*)	*b* (*SE*)	*b* (*SE*)	*b* (*SE*)	*b* (*SE*)	*b* (*SE*)	*b* (*SE*)	*b* (*SE*)
*Level 1*												
Individual status (IS)	.20*** (.05)	.14 (.09)	.12* (.06)	.05 (.10)	.22*** (.06)	.18^+^ (.11)	.18*** (.05)	.18^+^ (.10)	.35*** (.06)	.33*** (.08)	.23*** (.06)	.20** (.08)
*Level 2*												
Clique status	.19 (.11)	.19 (.11)	.07 (.12)	.05 (.10)	.25** (.10)	.25** (.10)	.10 (.10)	.10 (.10)	.07 (13)	.07 (13)	.21 (.14)	.21 (.14)
Clique size	−.02** (.01)	−.02** (.01)	−.01^+^ (.01)	.01 (.00)	−.01 (.01)	−.01 (.01)	.−00 (.01)	.−00 (.01)	−.00 (.00)	−.00 (.00)	.00 (.00)	−.00 (.00)
Clique hierarchization (CH)	.06 (.15)	.06 (.15)	.06 (.16)	−.01 (.16)	−.31* (.13)	−.31* (.13)	−.17 (.13)	−.17 (.13)	.34^+^ (.19)	.34^+^ (.19)	−.27 (.21)	−.27 (.21)
*Cross*-*level interaction*												
IS × CH		.81 (.85)		.81 (.92)		.36 (.75)		.00 (.68)		.27 (.64)		.45 (.62)
*df*	4	1	4	1	4	1	4	1	4	1	4	1
χ^2^ Deviance difference^b^	29.66***	.89	8.06^+^	0.76	26.48***	.23	13.67**	.00	52.10***	.18	18.94***	.52

^+^ *p* < .1; * *p* < .05; ** *p* < .01; *** *p* < .001

^a^For mixed-gender cliques we also included gender as control variable (girl = 0/boy = 1). The effect of gender was for all models −.15*** (.02)

^b^The decrease in χ^2^ deviance for models 1 of boys’ and girls’ cliques is compared with the deviance of the empty model, and of mixed-gender cliques compared with the deviance of the model including only gender. Model 2 is compared with model 1

**Fig. 2:**
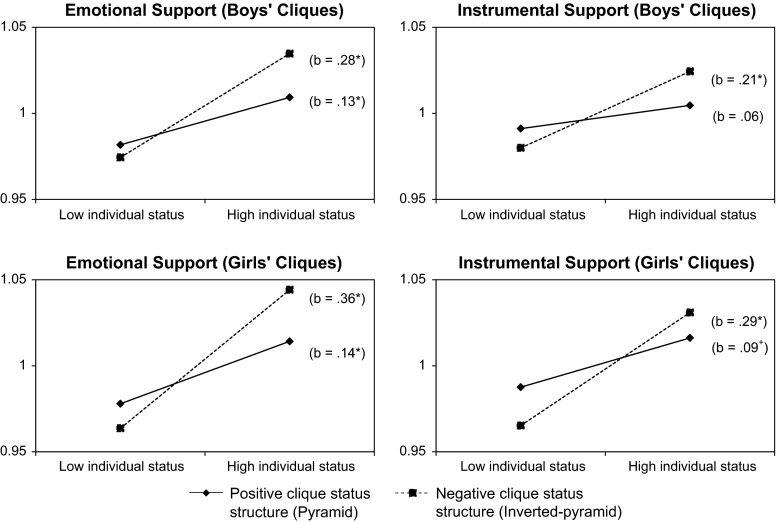
Simple slopes between individual status and emotional support and instrumental support in boys’ cliques and girls’ cliques for positive and negative clique status structure (*p < .05)

Again, models with clique status structure were similar to those with clique hierarchization (Models 1; Table 5). However, in the models with clique status structure the relationship between clique status and emotional support was positive for boys’ cliques. Furthermore, we found a negative main effect of clique status structure in mixed-gender cliques for emotional and instrumental support, suggesting that prosocial behavior was higher in cliques where the clique status structure reflected an inverted pyramid (i.e., more high status adolescents within the clique than low status adolescents). With regard to our hypotheses, clique status structure moderated the relationship between individual status and prosocial behavior (both emotional and instrumental support) in boys’ and girls’ cliques (Models 2). Contrary to our expectations, in these cliques individual status was particularly related to emotional support when the clique status structure followed an inverted pyramid shape pattern with a majority high status adolescents on the top and a minority of low status peers (see Fig. 2).

**Table 5:** Multilevel models of individual status and clique status structure for emotional and instrumental support

	Boys’ cliques				Girls’ cliques				Mixed-gender cliques^a^			
	Emotional support		Instrumental support		Emotional support		Instrumental support		Emotional support		Instrumental support	
	Model 1	Model 2	Model 1	Model 2	Model 1	Model 2	Model 1	Model 2	Model 1	Model 2	Model 1	Model 2
	*b* (*SE*)	*b* (*SE*)	*b* (*SE*)	*b* (*SE*)	*b* (*SE*)	*b* (*SE*)	*b* (*SE*)	*b* (*SE*)	*b* (*SE*)	*b* (*SE*)	*b* (*SE*)	*b* (*SE*)
*Level 1*												
Individual status (IS)	.16*** (.05)	.21*** (.05)	.09^+^ (.05)	.14* (.06)	.22*** (.06)	.26*** (.06)	.18*** (.05)	.21*** (.05)	.35*** (.06)	.35*** (.06)	.23*** (.06)	.24** (.08)
*Level 2*												
Clique status	.23* (.11)	.23* (.11)	.11 (.11)	.11 (.11)	.07 (.07)	.07 (.07)	.00 (.07)	.00 (.07)	.06 (.09)	.06 (.09)	.01 (.10)	.01 (.10)
Clique size	−.02* (.01)	−.02* (.01)	−.01 (.01)	−.01 (.01)	−.01 (.01)	−.01 (.01)	−.00 (.01)	−.00 (.01)	.00 (.00)	.00 (.00)	.00 (.00)	.00 (.00)
Clique status structure (CSS)	−.09 (.24)	−.09 (.24)	−.04 (.24)	−.04 (.24)	−.07 (.14)	−.07 (.14)	.03 (.13)	.03 (13)	−.36* (.17)	−.36* (.17)	−.36* (.18)	−.36* (.18)
*Cross*-*level interaction*												
IS × CSS		−1.54* (.71)		−1.45^+^ (.77)		−1.68** (.62)		−1.41* (.56)		.22 (.54)		−.03 (.52)
*df*	4	1	4	1	4	1	4	1	4	1	4	1
χ^2^ Deviance difference^b^	168.49***	4.61*	177.71***	3.53^+^	21.00***	7.30**	11.79*	6.37*	53.62***	.16	21.17***	.00

^+^ *p* < .1; * *p* < .05; ** *p* < .01; *** *p* < .001

^a^For mixed-gender cliques we also included gender as control variable (girl = 0/boy = 1). The effect of gender was for all models −.15***(.02)

^b^The decrease in χ^2^ deviance for models 1 of Boys’ and Girls’ cliques is compared with the deviance of the empty model, and of mixed-gender cliques compared with the deviance of the model including only gender. Model 2 is compared with model 1

## Discussion

Peers, especially within cliques, become very important in adolescence (e.g., Brown, 2004; Gifford-Smith and Brownell 2003; Salkind 2008), because they offer a setting where adolescents spend time with close others, and find belongingness and support (e.g., Brown 1990; Ellis and Zarbatany 2007; Kwon and Lease 2007; Prinstein and La Greca 2002). Adolescent peer cliques can be identified in an important way by their social stance in the peer domain but, although members within cliques are quite similar, they also differ with regard to social status (e.g., Adler and Adler 1998; Closson 2009). Individuals in cliques may vary with regard to their individual social status, resulting in cliques’ being either more hierarchical (with large differences in social status between clique members) or more egalitarian (with small differences in social status between clique members). To date, much remains unknown about how these differences might affect behaviors of clique members.

This article, therefore, set out to examine differences in the hierarchical organization of peer relationships within cliques, more specifically in what way the relationship between adolescents’ individual status, and aggression and prosocial behavior, was dependent on the variation (i.e., standard deviation) in individual social status within cliques (i.e., clique hierarchization), and the structure of status scores within a clique by subtracting the clique status median from the mean (i.e., clique status structure), to capture different configurations of a hierarchy within cliques (pyramid shape, inverted pyramid, or equal distribution of social status scores). It was argued that adolescents generally strive for status, which would encourage them to maintain the status they have in a context where there is a lot of competition for status. Such competition should be mostly present in groups and contexts where differences in status are small. Accordingly, we expected that there would be a stronger relationship between individual status and aggression in egalitarian cliques and in cliques with more high status adolescents relative to low status peers (inverted pyramid shape), because competition for status is likely to be higher in these cliques. Partially in line with these expectations, we found a moderating effect of clique status structure, but not clique hierarchization, on the relationship between individual status and physical and relational aggression in girls’ cliques. The results with clique status structure showed a consistent pattern in girls’ cliques that the relationship between adolescents’ status and their aggressive behavior appeared stronger when they resided in cliques that were not hierarchically organized.

Furthermore, we expected that, in more hierarchical cliques and in cliques with less high status adolescents relative to low status peers (pyramid shape), an individual’s status would be more strongly related to prosocial behavior, because in those cliques there is less competition for status, and thus more room for prosociality. However, we found evidence that individual status is actually more strongly related to prosocial behavior in boys’ and girls’ cliques with more high status adolescents relative to low status peers. It appeared that adolescents in cliques with a clique status structure that showed an inverted pyramid shape were perceived to be more aggressive, but also more cooperative than adolescents in hierarchies.

The underlying mechanisms that could explain these findings might be found when considering to whom the behaviors are directed. Previous research has shown that conflicts between groups can actually further strengthen in-group relations, specifically in situations where groups compete over resources and power (e.g., Brewer 1999; Sherif and Sherif 1953). Hierarchical groups could benefit from being aggressive towards members of other cliques instead of being aggressive towards clique members, not only to gain resources, but also to maintain the “good natured”, hierarchical structure within their own clique. In more egalitarian cliques however, aggression might be used as we argued before, towards clique members to compete over resources, power, or leadership within the clique. With regard to prosociality, behavior might actually be mostly directed toward clique (in-group) members to hold well-balanced relationships within the clique. Research has shown that cliques are often characterized by an environment that offers connectedness, acceptance, and support (Hartup 1993; Kwon and Lease 2007; Prinstein and La Greca 2002; Savin-Williams and Berndt 1990). Hence, hierarchical cliques might experience less conflict within their clique, but also have a less cohesive atmosphere, while egalitarian cliques might offer more of a “safe-haven” with occasional clashes over status.

We also found some gender-related nuances of the main findings. For girls’ cliques, we found significant effects of clique status structure on the relationship between individual status and aggression, but not for boys’ and mixed-gender cliques. This finding is partly surprising, because, although relational aggression is often found to be higher for girls than for boys, physical aggression is often found to be more prominent for boys than girls (e.g., Dijkstra et al. 2009; Hyde 1984; Pellegrini and Archer 2005; Rose et al. 2004; Vaillancourt and Hymel 2006). Also, boys’ interactions are often part of dominance hierarchies where aggression plays an important role (Geary et al. 2003). Furthermore, the expression of aggression also differs for boys and girls. Boys’ aggression and conflicts are often less disruptive of ongoing group activity, boys reconcile after a fight more quickly than girls, and are more likely to shrug off maltreatment by other boys, whereas girls are more likely to become upset by aggressive acts of others, and aggression tends to be expressed more in close relationships instead of in the larger peer group (e.g., Crick et al. 1999; Moffitt et al. 2002; Putallaz and Bierman 2004; Underwood 2003). Also, because girls are more likely to form close relationships with a fewer number of other girls, they are more likely to be sensitive to rejection, because when they are rejected, they have very few others or no one else with whom they (can) have a close relationship. It is, thus, possible that the structure within a clique can have a greater effect on the status-aggression relationship of girls than boys, because aggressive acts towards clique members have more severe consequences for girls than for boys. This might explain why we found a significant moderating effect of clique status structure for girls’ but not boys’ cliques. With regard to mixed-gender cliques, it is possible that there is more competition for status in same-gender than in mixed-gender cliques. For example, it has been argued that conflicts between same-sex adolescents are more common than between opposite-sex peers when it comes to resource control, for example, to attract the opposite sex (Pellegrini and Long 2003). This might explain why we found no effect of hierarchy structure on the relationship between individual status and aggression in mixed-gender cliques.

Furthermore, in boys’ and girls’ cliques, we found significant effects of clique status structure on the relationship between individual status and prosocial behavior, but not for mixed gender cliques. It is possible that mixed-gender cliques are inherently different from same gender cliques. For example, prosocial behavior in itself is more likely to occur between same-sex rather than other-sex peers, because needs are more easily recognized and communication is more effortless between individuals who are similar to each other (Byrne 1971; McPherson et al. 2001). Adolescents in mixed-gender cliques are likely to have fewer same-sex others who they would ask for help, and considering a hierarchy would only further diminish the number of possible others. It might be the case that the presence or absence of a hierarchy no longer matters in mixed-gender cliques, because the number of individuals one would ask for or give help is already very low.

It appears that different processes take place within mixed-gender cliques compared to same-gender cliques with regard to aggression and prosocial behavior, however, in order to draw clear conclusions on the associations between our variables of interest, the results need to be thoroughly replicated in future studies. Thus, studying (differences between same- and) mixed-gender cliques might be especially interesting for future research.

### Strengths, Limitations, and Directions for Future Research

The main strength of our study lies in the fact that we demonstrated that it is important not only to compare differences between individuals and cliques of adolescents, but also to take into account the internal structures of adolescent peer cliques. To better understand behavior of adolescents, it is shown that the internal hierarchy of peer relationships within cliques can affect behavioral outcomes of its members in different ways. In this respect, we introduced the status structure hierarchy as a new measure of hierarchy within groups. Results of this study indicate that this approach is particularly fruitful as it reveals to impact how social status is related to behavior in groups. Our analyses showed that the standard deviation does not appear to be an informative measure of configurations or structures of hierarchies, and one should consider what the effect is if standard deviation is used as a measure of hierarchy. Furthermore, this study showed gender clique specific findings that warrants a closer look in future research.

One limitation of our study is that we did not examine to whom the behaviors were directed. Directionality of behaviors might explain why we found the relationship between status and both aggression and prosocial behavior to be stronger in non-hierarchical cliques as mentioned before. Related to this, obtaining observations of behaviors other than those reported by classmates, or using more items, could also give more insight into the relationships between adolescent behaviors. It is possible that cliques are formed at grade or even school level, so that relationships with clique members transcend the classroom. This might also account for the fact that twenty percent of adolescents in our sample did not belong to a clique. Furthermore, although peer nominations are generally considered as reliable measures of behavior, as it is based on multiple informants (see for instance Veenstra et al. 2007), the usage of one item can be considered a limitation. A next step in research would be to untangle to whom aggression and prosociality is directed, within or across group boundaries, whether these behaviors are exhibited more by higher status or lower status adolescents (do they occur more top-down or bottom-up), and to what extent this differs for hierarchical and egalitarian groups. Future research should also focus on collecting data across classes and grades when studying cliques or adolescent peer relations.

Another limitation of our study is that the data were cross-sectional. We could, therefore, not draw any conclusions of causality of clique hierarchization and clique status structure on aggression and prosociality. Longitudinal data could give more insight into a possible causal relation. Related to this, longitudinal data could also deal with the idea that differences in relationships and behavior can be the consequence of specific selection and socialization processes (Veenstra et al. 2013). For example, aggressive adolescents might have higher status orientations and therefore choose friends who are relatively lower in status (creating a more hierarchical structure). In doing so they might need less aggression, because they compete less for status. However, cliques could also develop more hierarchically or more egalitarian over time due to clique members mimicking (social normative) behavior of others in their clique. In more egalitarian cliques for example, adolescents might copy aggressive behaviors, because they realize that this can lead to increasing ones’ status. In more hierarchical cliques however, clique members might observe that aggression is not part of the social norm, maybe even frowned upon, and members would therefore mimic other types of behavior. Longitudinal (social network) modeling (see Snijders et al. 2010) could give the opportunity to study selection and socialization processes as they happen over time, and would be a recommendation to use in future research.

### Conclusion

We found that the relationship between adolescents’ individual status and aggression and prosocial behavior, differs for different levels of clique status structure, and types of gender cliques. There appear to be different mechanisms at play *within* cliques when bearing in mind the internal structures of those cliques. Our results at the clique level further revealed that the standard deviation might be less adequate as a measure for assessing hierarchies within cliques. It even further stresses the importance of the clique context and taking into account internal clique structures when considering adolescent aggressive and prosocial behaviors. Recommendations for future research would, therefore, be to carefully consider which context is under study and which factors need to be taken into account with regard to that context. For example, contemplating directionality of behaviors and differences between same- and mixed-gender groupings would be a very interesting next step in adolescent research. Recognizing the importance of cliques and their characteristics can help us better understand why adolescents display aggressive and prosocial behaviors, and how internal group dynamics might facilitate or inhibit these behaviors.

## Figures and Tables

**Fig. 3:**
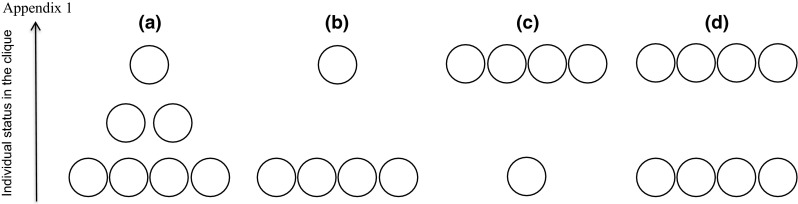
Example of possible configurations of clique hierarchies: pyramid (**a**, **b**), inverted pyramid (**c**), symmetric (**d**)

## Appendix 1

See Fig. 3.

## Appendix 2

See Table 6.

**Table 6:** Descriptive statistics of adolescents not in a clique and adolescents in cliques

	Adolescents not in a clique (*N* = 638)	Adolescents in cliques (*N* = 2674; *N*_cliques_ = 534)	Difference	
	Mean (SD)	Mean (SD)	t (*df*)	p
Gender (boy = 1)	.52^a^	.50^a^	.82 (3310)	.41
Individual status	.08 (.10)^b^	.11 (.13)^a^	−7.39 (3310)	<.01
Physical aggression	.09 (.17)^a^	.07 (.14)^b^	2.69 (3310)	<.01
Relational aggression	.11 (.13)^b^	.13 (.13)^a^	−2.62 (3310)	<.01
Emotional support	.10 (.08)^b^	.16 (.11)^a^	−15.48 (3310)	<.01
Instrumental support	.13 (.10)^b^	.21 (.11)^a^	−17.08 (3310)	<.01

Means in the same row that do not share superscripts differ at *p* < .05 in the Bonferroni test

## Appendix 3

See Table 7.

**Table 7:** Correlations between individual status, clique status, clique size, clique hierarchization, clique status structure, and the behavioral outcomes, for boys’ and girls’ cliques

	1	2	3	4	5	6	7	8	9
1. Individual status		.15**	.13**	.13**	.07*				
2. Physical aggression	.21**		.14**	−.08**	−.11**				
3. Relational aggression	.14**	.30**		−.01	−.02				
4. Emotional support	.09**	−.00	−.04		.55**				
5. Instrumental support	.05	−.04	−.02	.54**					
6. Clique status							.12**	.71**	.11**
7. Clique size						.12**		.25**	.14**
8. Clique hierarchization						.73**	.11**		.50**
9. Clique status structure						−.04	.04	.30**	

Boys’ cliques above and girls’ cliques below the diagonal (* *p* < .05; ** *p* < .01)

## Appendix 4

See Table 8.

**Table 8:** Correlations between individual status, clique status, clique size, clique hierarchization, clique status structure, and the behavioral outcomes, for mixed-gender cliques

	1	2	3	4	5	6	7	8	9
1. Individual status		.23**	.24**	.23**	.19**				
2. Physical aggression	.15**		.31**	−.07	−.07				
3. Relational aggression	.36**	.25**		−.00	.02				
4. Emotional support	.20**	−.10	−.02		.47**				
5. Instrumental support	.07	−.26**	−.07	.53**					
6. Clique status							.06	.78**	−.24**
7. Clique size						.08		.06	.15**
8. Clique hierarchization						.76**	.07		.05
9. Clique status structure						−.28**	.15**	.07	

Boys above and girls below the diagonal (** *p* < .01)
